# Supplementary material for: Insight into the Postbiotic Potential of the Autochthonous Bacteriocin-Producing Enterococcus faecium BGZLM1-5 in the Reduction in the Abundance of Listeria monocytogenes ATCC19111 in a Milk Model
Source: Microorganisms. 2023 Nov 23;11(12):2844. doi: 10.3390/microorganisms11122844 (PMC10745621; doi:10.3390/microorganisms11122844)
Supplement: Supplementary file 1 [file microorganisms-11-02844-s001.zip › microorganisms-2667805-supplementary.pdf]

Supplementary Table 1. Putative antimicrobial resistance genes in *Enterococcus faecium* BGZLM1-5.

Antibiotic	Gene	Alignment Length/Gene Length	Contig	Position in contig	Identity	Accession number
Macrolides	*msrC*	1479/1479	NODE_3_length_98575_cov_51.187084	82311..83789	98.9182	AY004350
Aminoglycosides	*aac(6')-Ii*	549/549	NODE_29_length_33826_cov_40.384997	19036..19584	99.6357	L12710
Disinfectant	*ClpL*	2115/2115	NODE_55_length_15392_cov_159.710514	82311..83789	99.2908	CP023753

Note: Selected %ID threshold for ResFinder: 90 %; Selected minimum length for ResFinder: 60%

Supplementary Table 2. Putative virulence factors in *Enterococcus faecium* BGZLM1-5.

VFclass	Virulence factors	Related genes	***Enterococcus faecium* BGZLM1-5 (Prediction)**
			**draft (draft)**
Adherence	Ebp pili	*ebpA*	orf00624; orf00625; orf02460
		ebpB	orf00623
		ebpC	orf00622
		srtC	orf00621
	EcbA	*ecbA*	orf01267
	EfaA	*efaA*	orf01774; orf02610
	SgrA	*sgrA*	orf01406
Antiphagocytosis	Capsule	*cpsA/uppS*	orf01658
		cpsB/cdsA	orf01657
Biofilm formation	BopD	*bopD*	orf01915
Immune evasion	Capsule(Streptococcus)	-	orf02634
Enzyme	Serine-threonine phosphatase(*Listeria*)	*stp*	Orf00250
Iron uptake	Periplasmic binding protein-dependent ABC transport systems(Vibrio)	*vctC*	Orf00050
Surface protein anchoring	Lipoprotein diacylglyceryl transferase(Listeria)	*igT*	orf00792

Supplementary Table 3. Putative plasmids identification against a gram-positive database using PlasmidFinder of *Enterococcus faecium* BGZLM1-5.

Database	Plasmid	Query / Template length	Contig	Position in contig	Note	Accession number
Rep3	rep18a	933/933	NODE_59_length_13371_cov_169.066445	1118..2050	repA(p200B)	AB158402
	rep29	738/738	NODE_83_length_6222_cov_290.081214	4751..5488	ORF8(pCIZ2)	DQ832184
Inc18	rep2	1494/1494	NODE_79_length_7592_cov_226.482786	4272..5765	orf1(pRE25)	X92945
RepA_N	repUS15	1041/1041	NODE_20_length_43538_cov_49.149801	39121..40161	repA(pNB2354p1)	CP004064

Supplementary Figure 1. The presence of genes encoding bacteriocins and non-bactericidal post-translationally modified peptides using the BAGEL4 online tool.

A)

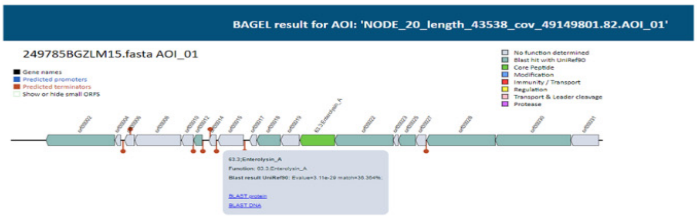
BAGEL result for AOI: 'NODE_20_length_43538_cov_49149801.82.AOI_01'
249785BGZLM15.fasta AOI_01

B)

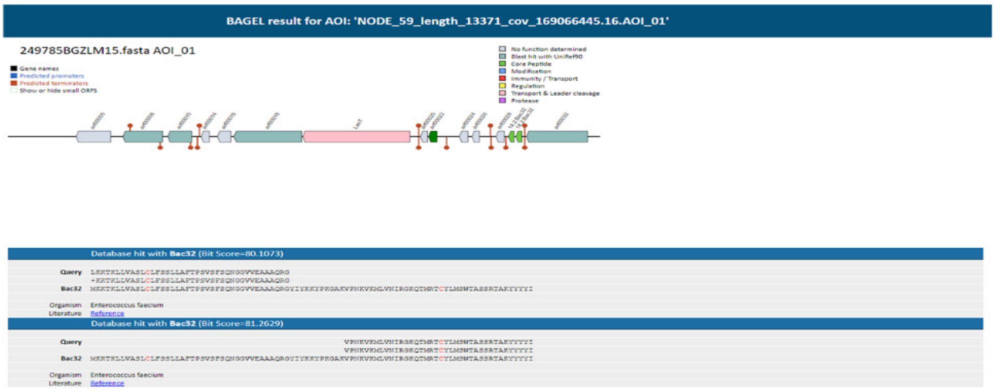
BAGEL result for AOI: 'NODE_59_length_13371_cov_169066445.16.AOI_01'
249785BGZLM15.fasta AOI_01
Database hit with Bac32
Organism Enterococcus faecium

C)

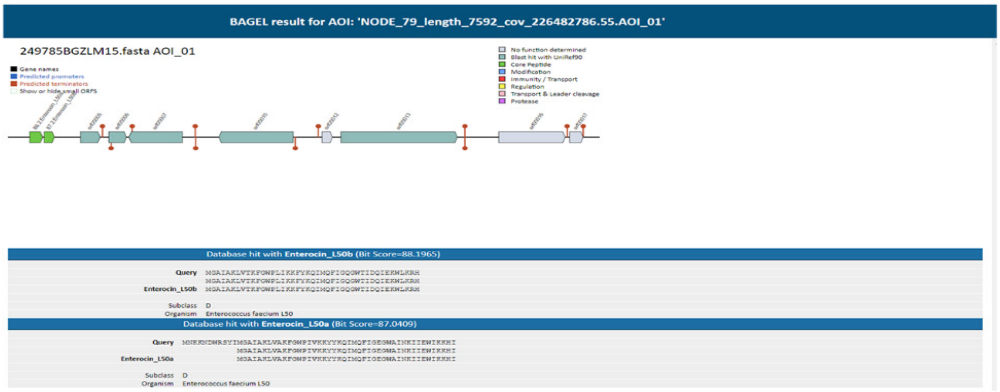
BAGEL result for AOI: 'NODE_79_length_7592_cov_226482786.55.AOI_01'
249785BGZLM15.fasta AOI_01
Database hit with Enterocin_L50b (Bit Score=88.1965)
Database hit with Enterocin_L50a (Bit Score=87.0409)

D)

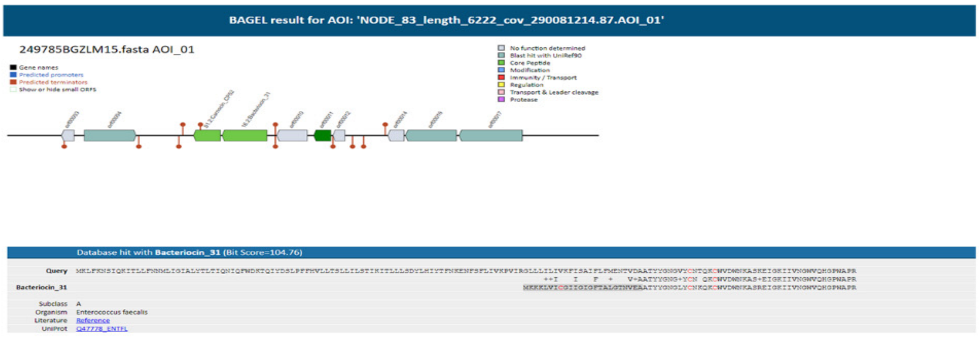
BAGEL result for AOI: 'NODE_83_length_6222_cov_290081214.87.AOI_01'
249785BGZLM15.fasta AOI_01
Database hit with Bacteriocin_31 (Bit Score=104.76)
